# Evaluation of different suspicion indices in identifying patients with Niemann-Pick disease Type C in clinical practice: a post hoc analysis of a retrospective chart review

**DOI:** 10.1186/s13023-019-1124-3

**Published:** 2019-07-02

**Authors:** Mercedes Pineda, Katarína Juríčková, Parvaneh Karimzadeh, Miriam Kolniková, Věra Malinová, Juan Torres, Stefan A. Kolb

**Affiliations:** 1 Neuropediatrics, Institut Pediatric Hospital Sant Joan de Déu, Barcelona, Spain; 2 Department of Pediatrics, Centre for Inborn Errors of Metabolism, Comenius University Medical School and National Institute of Children’s Diseases, Bratislava, Slovakia; 3 Department of Paediatric Neurology, Paediatric Neurology Research Centre, Shahid Beheshti University of Medical Sciences, Mofid Children Hospital, Tehran, Iran; 4 Department of Pediatric Neurology, Comenius University Medical School and National Institute of Children’s Diseases, Bratislava, Slovakia; 5 Department of Pediatrics and Adolescent Medicine, First Faculty of Medicine, Charles University and General University Hospital, Prague, Czech Republic; 6 Syntax for Science S.L, Palma de Mallorca, Spain; 7 Actelion Pharmaceuticals Ltd., a Janssen Pharmaceutical Company of Johnson & Johnson, Allschwil, Switzerland

**Keywords:** Niemann-Pick disease Type C, NP-C, Clinical diagnosis, NP-C disability scales, NP-C Suspicion Index, Screening, Neurologic findings, Patient detection, Hepatosplenomegaly, Neonatal jaundice

## Abstract

**Background:**

Niemann-Pick disease Type C (NP-C) is a lysosomal lipid storage disorder with varying symptomatology depending on the age of onset. The diagnosis of NP-C is challenging due to heterogeneous nonspecific clinical presentation of the disease. NP-C Suspicion Index (SI) was developed to aid screening and identification of patients with suspicion of NP-C for further clinical evaluation. Here we assess the performance of five NP-C SI models to identify patients with NP-C compared with clinical practice to determine the best SI model for identification of each clinical form of NP-C by age.

**Methods:**

This was a post hoc analysis of a retrospective chart review of patient data collected from five expert NP-C centers. The study assessed the proportion of patients with NP-C who could have been identified using the Original SI, Refined SI, 2/7 SI, 2/3 SI, and Early-Onset SI and evaluated the performance of each SI against clinical practice. A score above a threshold of 70 points for the Original SI, 40 points for the Refined SI, 6 points for the Early-Onset SI, and 2 points for the 2/7 and 2/3 SIs represented identification of NP-C.

**Results:**

The study included 63 patients, and of these, 23.8% had a family history of NP-C. Of the available SI tools, the Refined SI performed well in identifying patients with NP-C across all age groups (77.8% infantile, 100% juvenile and 100% adult groups), and earlier identification than clinical diagnosis would have been possible in 50.0% of infantile, 72.7% of juvenile and 87.0% of adult patients. Patients who were not detected by the Refined SI prior to clinical diagnosis mainly presented with delayed developmental milestones, visceral manifestations, neurologic hypotonia, clumsiness, ataxia, vertical supranuclear gaze palsy, parent or siblings with NP-C, dysarthria/dysphagia and psychotic symptoms.

**Conclusion:**

This study demonstrated the applicability of various SI models for screening and identification of patients with NP-C for further clinical evaluation. Although NP-C is rare and the patient population is limited, this study was conducted in a real-world setting and confirms SI models as useful screening tools that facilitate identification of patients with NP-C earlier in their disease course.

**Electronic supplementary material:**

The online version of this article (10.1186/s13023-019-1124-3) contains supplementary material, which is available to authorized users.

## Background

Niemann-Pick disease Type C (NP-C) is a fatal, autosomal-recessive lysosomal lipid storage disease with a wide spectrum of clinical presentations [1, 2]. The onset of NP-C ranges from the perinatal period to adulthood, with varying symptomatology depending on the age of onset. Patients who develop NP-C during early infancy frequently present with visceral manifestations such as splenomegaly, hepatomegaly, neonatal jaundice, and hyperbilirubinemia, with varying degrees of neurologic signs [3, 4]. Adolescent or adult onset of NP-C presents with varying combinations of progressive neurologic deficits, e.g. ataxia, dystonia, and/or dementia, vertical supranuclear gaze palsy (VSGP), or major psychiatric illness [4]. Due to the heterogeneous and nonspecific clinical presentation of the disease, the diagnosis of NP-C may be challenging and often results in significant diagnostic delays [5, 6].

Miglustat (Zavesca®, Actelion Pharmaceuticals Ltd.) is the only disease-specific therapy approved to treat neurologic manifestations of NP-C and has been shown to delay disease progression and stabilize certain symptoms of the disease [6–8]^1^. Patients who receive treatment early during the disease course seem to have better prognosis and improved clinical outcomes, highlighting the need for early diagnosis and treatment initiation for NP-C [9–11].

To aid the identification of patients with a suspicion of NP-C for subsequent clinical diagnosis, the NP-C Suspicion Index (SI) was developed, in which NP-C signs and symptoms were categorized into visceral, neurologic, and psychiatric domains [12]. The model was effective in identifying NP-C in patients at or above the age of 4 years [4], but not in pediatric patients below 4 years of age. For these patients a separate Early-Onset NP-C SI has been developed [3]. Hendriksz et al. further refined the Original SI, into the Refined SI, which utilized the predictive power of both the individual symptoms and combinations of individual symptoms [13]. These analyses also resulted in a simple 2 out of 7 scoring model (2/7 SI) for daily and quick use that takes into account the combination of symptoms and assigns a high suspicion score to patients who present with either two of seven key symptoms or VSGP alone [13]. Furthermore, the 2 out of 3 SI (2/3 SI) was developed to aid identification of NP-C in patients with early onset ataxia [14].

Here we assess the performance of the five NP-C SI models (Original SI, Refined SI, 2/7 SI, 2/3 SI, and Early-Onset SI) to identify patients with NP-C compared with clinical practice, and whether the SI models can identify NP-C earlier than in clinical practice. The most performant SI to use for the identification of each clinical form of NP-C, as defined by the infantile, juvenile, or adult age groups, is also determined.

## Methods

### Study design and population

This was a post hoc analysis of a retrospective, observational chart review of patient data collected between February and December 2016 from five expert NP-C centers. Details of this patient cohort have been described previously [15]. In brief, eligible patients had a confirmed diagnosis of NP-C by classical filipin staining with or without the presence of two known pathogenic *NPC1*/*NPC2* mutations, or variant filipin staining with the presence of *NPC1*/*NPC2* mutations, or the presence of *NPC1*/*NPC2* mutations. Patients with lysosomal storage diseases or enzyme deficiency diseases other than NP-C and a variant filipin staining without confirmatory genetic diagnosis of NP-C by two confirmed known *NPC* mutations were excluded. The participating site or physician was responsible for obtaining ethical approval. Informed consent was obtained either from the patient or their parents/legal guardians according to local laws. Patients were subcategorized by the age of clinical diagnosis into infantile (< 4 years), juvenile (≥ 4–< 16 years), and adult (≥16 years) groups.

### Study endpoints

The analyses were performed to determine the proportions of patients who could have been identified with NP-C using Original SI [12], Refined SI [13], 2/7 SI [13], 2/3 SI [14], and Early-Onset SI [3]. This study evaluated the following: the proportion of patients for whom the assessed SI could have identified patients with NP-C earlier than and at the same time as the clinician, the proportion of patients for whom the assessed SI could not have identified patients with NP-C as quickly as the clinician, and the years gained with each SI versus clinical practice. Based on these outcomes, the most appropriate model was determined using the risk prediction score to identify patients with NP-C for infantile, juvenile and adult patients.

### Data analyses

The analysis population included all patients who were in the database (*n* = 63). The date of onset of clinical signs and symptoms was noted from each patient’s medical history. Each time a new clinical sign or symptom was noted in the medical history, the scores for each SI model were re-calculated, resulting in an increasing cumulative score for each SI for each patient over time. A score above the threshold score indicating high suspicion of NP-C for each SI model, namely a threshold of 70 points for the Original SI [12], above 40 points for the Refined SI [13], above 6 points for the Early-Onset SI [3] and above 2 points for the 2/7 [13] and 2/3 SIs [14] represented identification of NP-C (Additional file 1: Figure S1, Additional file 2: Figure S2 and Additional file 4: Table S1). Diagnostic procedures were undertaken according to local clinical practices, and may vary between centers. As the SI symptom of “parent or siblings with NP-C” does not have a date of onset per se, the date for this feature was set to the day of clinical diagnosis. Symptoms occurring within 30 days of actual clinical diagnosis were given the same date as the clinical diagnosis to reflect time needed to conduct diagnostic tests and subsequent interpretation of the results by a clinician. Patients with NP-C identified using SI models within 30 days of actual clinical diagnosis were given the same date as the clinical diagnosis to prevent statistical bias due to short time differences.

### Statistical analyses

For each SI model, the percentage of patients who crossed the threshold score for high-risk prediction was calculated to identify two subpopulations: those where the SI model could detect patients with NP-C and those where it could not. For the subpopulation where the SI model could detect patients with NP-C, the time difference between identification of each patient by the SI models and actual clinical diagnosis was calculated (age at medical diagnosis – age at SI model threshold crossing = difference in years). The proportions of patients in whom the SI model could identify NP-C earlier than clinical diagnosis, at the same time as the clinician, or later than the clinician were calculated. The annual rate of SI score increase was estimated using regression analysis of the NP-C SI scores of each patient and each SI model. Average slopes (and 95% confidence intervals [CI]) for each patient age group were calculated for each of the SI models. Descriptive statistics were provided for patients diagnosed earlier or later than clinical diagnosis by each SI model.

## Results

### Patient population

A total of 63 patients were included in this retrospective analysis. The clinical and treatment characteristics of patients have been described previously [15]. In brief, the analysis included 37 male and 26 female patients. A quarter of these patients (23.8%) had a family history of NP-C. The majority of patients (82.5%) received miglustat therapy with a median (range) duration of 2.89 (0.01–9.7) years, and 61.9% of patients received treatment for more than 1 year.

Patients were grouped based on age at diagnosis into infantile (*n* = 18), juvenile (*n* = 22), and adult (*n* = 23) groups. The time between onset of neurologic symptoms and diagnosis varied across the groups; it was greater in the adult-onset group compared with the infantile- and juvenile-onset groups. The mean (standard deviation [SD]) age at first presentation of neurologic symptoms were 2.28 (4.30) years for the infantile group, 9.46 (4.43) years for the juvenile group, and 17.81 (8.69) years for the adult groups. The mean age at diagnosis for the infantile, juvenile and adult groups were 2.15 (1.10) years, 10.97 (3.82) years and 25.98 (8.15) years, respectively.

### NP-C identification: comparison between NP-C SI models and clinical practice

#### Original SI versus clinical diagnosis

**Fig. 1:**
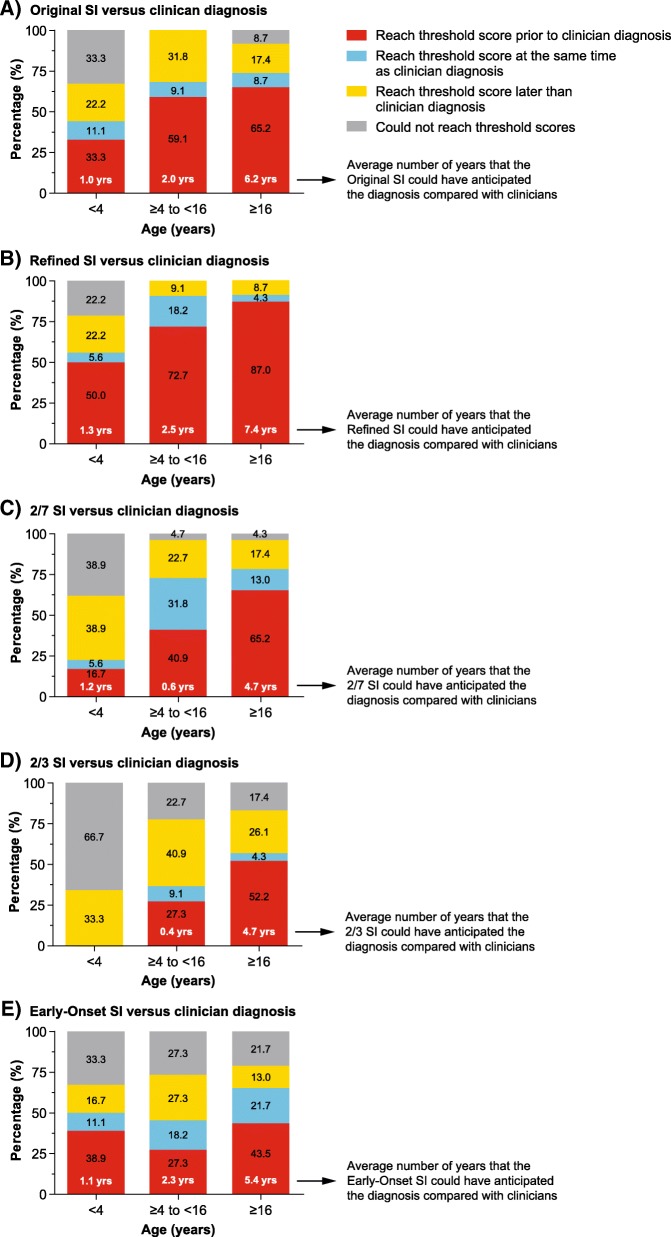
Pairwise comparisons of different SIs with clinician diagnosis. SI, Suspicion Index

Overall, 66.7% of infantile, 100% of juvenile, and 91.3% of adult patients diagnosed with NP-C by clinicians would have been identified by the Original SI as being of high suspicion of NP-C. The Original SI would have resulted in earlier identification of NP-C in 33.3% of infantile, 59.1% of juvenile, and 65.2% of adult patients (Fig. 1a). Using the Original SI, the mean (SD) number of years gained to identify patients with a suspicion of NP-C compared with clinician were 1.0 (0.8), 2.0 (2.6), and 6.2 (5.6) years for infantile, juvenile, and adult patients, respectively. The Original SI would have identified NP-C at the same time (± 30 days) as clinical diagnosis in 11.1% of infantile, 9.1% of juvenile, and 8.7% of adult patients.

#### Refined SI versus clinical diagnosis

The Refined SI would have identified 77.8% of infantile, 100% of juvenile, and 100% of adult patients diagnosed with NP-C by the clinician as being of high suspicion of NP-C. The Refined SI would have resulted in earlier identification of NP-C in 50.0% of infantile, 72.7% of juvenile, and 87.0% of adult patients (Fig. 1b). Using the Refined SI, the mean (SD) number of years gained to identify patients with a suspicion of NP-C compared with clinician were 1.3 (0.9), 2.5 (2.8), and 7.4 (5.7) years for infantile, juvenile, and adult patients, respectively. The Refined SI would have identified NP-C at the same time as the clinician in 5.6% of infantile, 18.2% of juvenile, and 4.3% of adult patients (Fig. 1b).

**Fig. 2:**
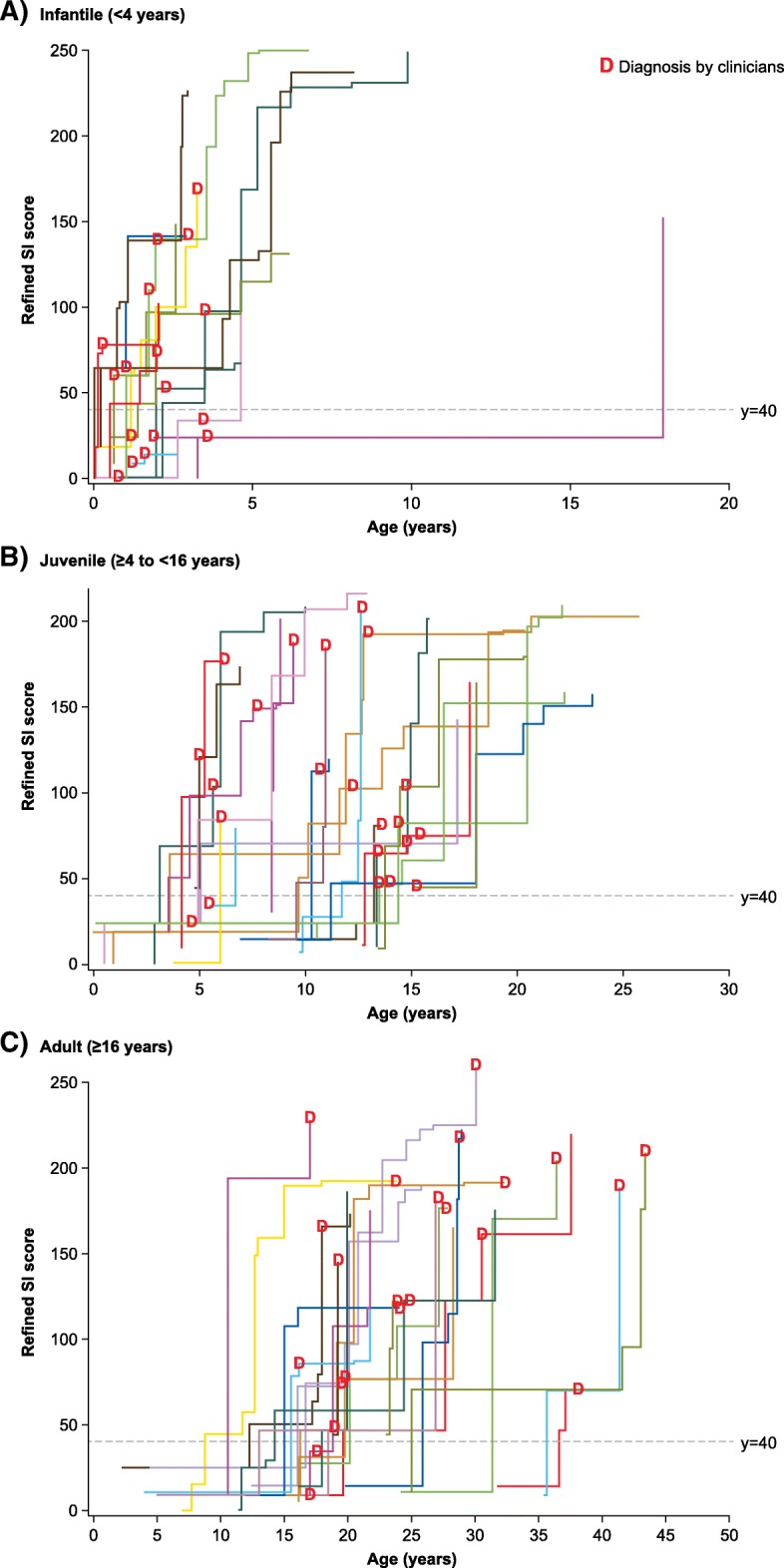
Individual patients’ scores for the Refined SI versus clinician diagnosis. Patients’ SI scores increase every time the clinical signs or symptoms occur. The SI threshold is shown as a dotted horizontal line, and the time of medical diagnosis is represented by a red “D”, for visual purposes**.** SI, Suspicion Index

The individual cumulative patients’ scores for the Refined SI increased over time in all age groups (Fig. 2). The first possible clinical diagnosis of NP-C varied across the groups, and more patients in the infantile group than in the juvenile and adult groups would have been diagnosed with NP-C by the clinician than by using the Refined SI. Overall, 8/18 (44.4%) patients in the infantile group, 2/22 (9.1%) patients in the juvenile group and 2/23 (8.7%) patients in the adult group would have been diagnosed by the clinician earlier than by using the Refined SI (Fig. 2).

#### 2 out of 7 SI versus clinical diagnosis

Overall, 61.1% of infantile, 95.5% of juvenile, and 95.7% of adult patients diagnosed with NP-C by the clinician would have been identified with a high suspicion of NP-C using the 2/7 SI. The 2/7 SI would have resulted in earlier identification of NP-C in 16.7% of infantile, 40.9% of juvenile, and 65.2% of adult patients (Fig. 1c). The average (SD) number of years gained to anticipate NP-C earlier than the clinician were 1.2 (0.8), 0.6 (0.3) and 4.7 (4.8) years for infantile, juvenile and adult patients respectively. Overall, 5.6% of infantile, 31.8% of juvenile, and 13.0% of adult patients would have been identified with the 2/7 SI at the same time as the clinical diagnosis.

#### 2 out of 3 SI versus clinical diagnosis

The 2/3 SI would have identified 33.3% of infantile, 77.3% of juvenile, and 82.6% of adult patients with NP-C as being of high suspicion of NP-C. None of the patients in the infantile group would have been identified using the 2/3 SI prior to the clinical diagnosis, but 27.3% of juvenile patients and 52.2% of adult patients would have been identified earlier than the clinical diagnosis (Fig. 1d). This resulted in a mean (SD) gain of 0.0 (0.0), 0.4 (0.3), and 4.7 (4.5) years for infantile, juvenile, and adult patients, respectively. Using the 2/3 SI, none of the infantile patients, 9.1% of juvenile patients, and 4.3% of adult patients would have been identified with NP-C at the same time as the clinical diagnosis.

#### Early-onset SI versus clinical diagnosis

Overall, 66.7% of infantile, 72.7% of juvenile, and 78.3% of adult patients diagnosed with NP-C by clinicians would have been identified by the Early-Onset SI as being of high suspicion of NP-C. Earlier identification of NP-C would have been achieved in 38.9, 27.3, and 43.5% of infantile, juvenile, and adult patients, respectively, and at the same time as the clinician in 11.1% of infantile, 18.2% of juvenile, and 21.7% of adult patients (Fig. 1e). In patients who could have been identified earlier by the Early-Onset SI than by the clinician, the mean (SD) number of years gained with earlier identification were 1.1 (1.0), 2.3 (3.1), and 5.4 (4.1) years for infantile, juvenile, and adult patients, respectively.

### Annual rate of NPC SI score increase

Using the Refined SI, the mean (95% CI) annual rate of increase in SI scores was 37.0 (25.6, 48.5) for the infantile group, 29.3 (18.5, 40.1) for the juvenile group and 23.7 (9.0, 38.5) for the adult groups, although the differences were not statistically significant. Using the Original SI, the mean (95% CI) annual rate of increase in SI scores for the infantile (41.7 [24.4, 59.1]), and adult groups (15.6 [11.4, 19.8]) differed significantly (*p* < 0.05); the mean annual rate of SI score increase for the juvenile group was not significantly different to both other groups (29.1 [19.4, 38.8]).

### Symptoms in patients diagnosed in clinical practice earlier than, or at the same time as, the SI models

**Fig. 3:**
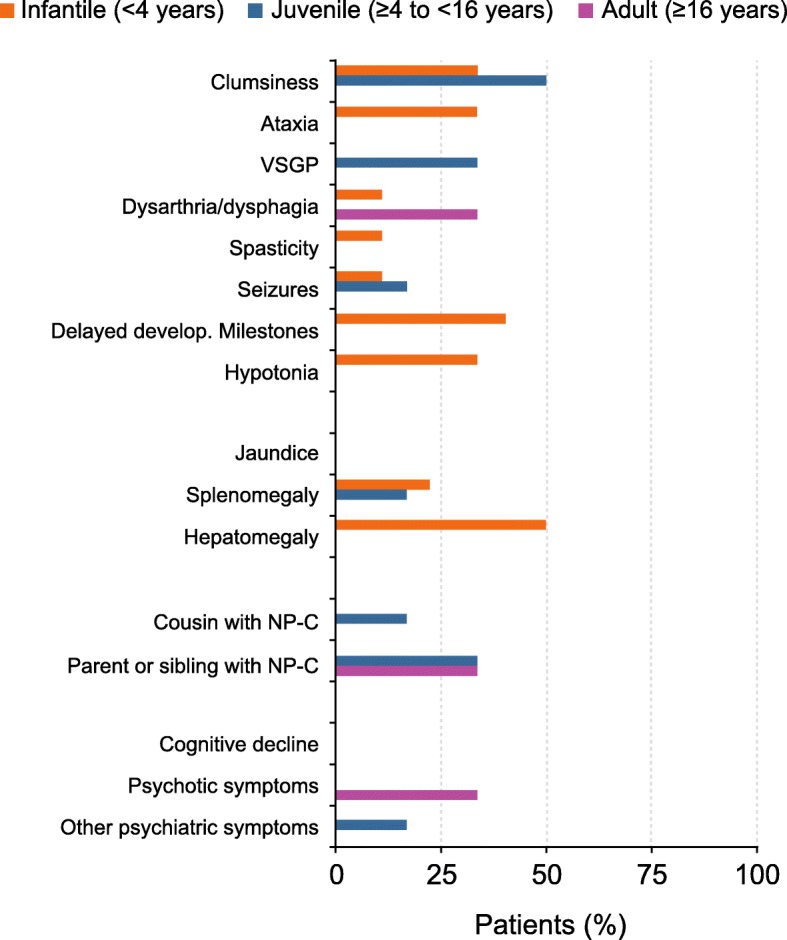
Symptoms in patients diagnosed at clinic earlier than or the same time as Refined SI. NP-C, Niemann-Pick disease Type C; SI, Suspicion Index; VSGP, vertical supranuclear gaze palsy

Infantile patients diagnosed earlier in clinical practice or at the same time as the Refined SI displayed frequent manifestations of delayed developmental milestones, hepatomegaly, ataxia, and hypotonia, but these symptoms were not frequent in older patients (Fig. 3). The majority of symptoms in the infantile group appeared across the visceral and neurologic domains, but the symptoms were less common in the psychiatric domain. The juvenile and adult patients presented with symptoms across all three domains with fewer manifestations in the visceral domain. VSGP and clumsiness were frequent in juvenile patients but not in adults, and having a family history (parent or sibling) of NP-C was equally likely in juvenile and adult patients. Dysarthria/dysphagia and psychotic symptoms were the most common symptoms not detected by the Refined SI.

The pattern of presenting symptoms for patients who were not identified with NP-C prior to using the Original SI (Additional file 3: Figure S3A) was similar to those observed with the Refined SI for infantile and juvenile patients, but with a higher frequency of visceral and neurologic manifestations in these groups. Adult patients presented with symptoms across all three domains, with the highest frequency of symptoms in the neurologic and psychiatric domains in the Original SI versus the Refined SI.

The pattern of presenting symptoms for the 2/7 SI, 2/3 SI, and Early-Onset SI (Additional file 3: Figure S3B, S3C, and S3D) were similar to the Refined SI in the infantile group; however, unlike with the use of the Refined SI, the majority of patients in the juvenile groups for these SIs displayed ataxia, splenomegaly, clumsiness, and VSGP during clinical diagnosis. The most common symptom not detected by the 2/7 SI in juvenile patients was cognitive decline, which did not trigger the risk prediction score of > 1. Having a parent or sibling with NP-C or psychotic symptoms was not commonly missed by the 2/7, 2/3, and Early-Onset SIs (Additional file 3: Figure S3B, S3C, and S3D). Adult patients not detected by these SIs most often presented with ataxia, clumsiness, cognitive decline, dysarthria/dysphagia, and VSGP.

## Discussion

The online NP-C SI screening models were developed to assist screening for patients with a high likelihood of NP-C for further clinical investigation and diagnostics. The current study assessed whether the existing SI models could identify patients with NP-C earlier than the clinical diagnosis. Of the available SI models, the Refined SI was found to be the best model for identifying patients with NP-C across all age groups. Using the Refined SI, earlier identification of NP-C would have been possible in 50.0% of infantile patients, 72.7% of juvenile patients, and 87.0% of adult patients as compared with a clinical diagnosis.

When compared with the Original SI, the Refined SI was able to identify an additional 11.1% of infantile, and 8.7% of adult patients with high suspicion of NP-C. Moreover, the Refined SI performed better than the Original SI, identifying an additional 16.7% of infantile, 13.6% of juvenile, and 21.8% of adult patients with NP-C earlier than the clinical diagnosis, and 9.1% of juvenile patients at the same time as the clinical diagnosis. The higher precision of the Refined SI compared with the Original SI observed in this study is largely in agreement with the literature, where it is reported that the Refined SI could predict 83% of patients with NP-C as compared with 71% using the Original SI [13]. The Early-Onset SI performed better than the Original SI in identifying patients below 4 years of age; however, it did not outperform the Refined SI. This may be because half of the patients in this study are > 2 years old [3, 15]. It may also be possible that some patients with mild symptoms may have been diagnosed before 4 years of age, but their symptoms may not have reached the high suspicion score threshold until after 4 years of age.

Some SI models were not able to identify certain patients with NP-C as quickly as the clinician, either because of the limited number of signs and symptoms, the presence of different symptoms to those included in these SIs, or the SIs being developed for specific patient/age groups. The 2/3 SI model was intended for rapid appraisal of suspected NP-C cases in unexplained early-onset ataxia, as patients with this condition are at high risk of having NP-C. Because the 2/7 model assesses only seven signs and symptoms of NP-C, it acts as a quick and easy screening tool in a clinic without access to the Internet. The 2/7 model should be used along with other models to provide a comprehensive picture of the disease and accurate screening for NP-C. The characteristic symptomatology included in the Early-Onset SI is specific to patients < 4 years of age and may not work as well as the Refined SI, which was designed for patients older than 4 years of age, in juvenile and adult patient populations [3].

As the NPC SI models assign scores upon the appearance of symptoms, and that scores will increase over time, the scales might be used to assess the rate of disease progression for individual or populations of patients. Only the Original SI and Refined SI were assessed as they are can be applied to each of the age groups, whereas the more specialised SI models are intended for use in more limited patient populations. Our data show that the Original SI discriminates between the rapid increase of score in infantile patients and the slower increase in score in adult patients, however, the Refined SI does not discriminate the rate of disease progression in different patient groups. It should be noted that as disease monitoring is not the intended use of the NPC SI models, that the NPC models only contain those symptoms that were considered discriminatory during their construction, and that they do not make any allowance for symptom severity; other disease specific scales [11, 16, 17] should instead be used for the monitoring of disease progression and response to treatment.

The SI tools considered only a certain set of symptoms or a combination of them, which can increase the predictive power of these tools in providing discriminatory evidence. As certain clinical signs commonly encountered in patients with NP-C are also seen in other types of disease, these symptoms do not have a higher associated score in the SIs. For example, ataxia, a prominent, early and frequent symptom of NP-C, is not included in the 2/7 SI due to its low specificity and poor discriminatory power between NP-C and non-NP-C when in combination with other symptoms. Recent evidence suggests that ocular motor abnormalities are some of the first indications of NP-C but are often subtle and overlooked at an early stage of the disease [6, 18]. Detailed examination by experts has shown that a high proportion of even very young patients have some degree of ocular impairment; therefore, a careful neuro-ophthalmological examination is crucial for the diagnosis of NP-C [6, 18].

Despite recent developments in NP-C diagnostics, a large proportion of patients with NP-C remain undiagnosed due to the limited awareness of the disease and the limited ability of diagnosing physicians to link often non-specific symptoms with NP-C [19]. The NP-C SI models are great educational tools for increasing disease awareness among physicians, and contribute to a vital link in identifying suspected cases of NP-C between clinical observations and eventual laboratory confirmation. In a certain number of patients, it may not be possible to identify NP-C using these SI models due to mild and atypical symptomatology; screening studies are necessary to detect NP-C in these patients. However, there are still numerous undetected patients with a clear, detectable clinical presentation, and SI models are valuable for advancing diagnosis in these patients, allowing earlier treatment and subsequent improvement in prognosis.

The outcomes of this analysis are highly valuable considering the available evidence on the applicability of different SI models for use in clinical practice. The time saved by 1 year for possible earlier identification of NP-C in infantile patients is large considering the rapidly progressive infantile NP-C phenotype and can have a profound difference in long-term outcomes; earlier identification may result in earlier intervention and thus can have a more pronounced clinical effect [9–11].

Because NP-C is a rare disease, the patient population is limited and obtaining a sufficient sample size is difficult. Due to the small sample size, the patient cohort may not be fully representative of the NP-C population as a whole. The retrospective nature of the data used for this analysis makes it difficult to assess the usefulness of these SIs in helping with the early detection of NP-C and predicting disease progression in these patients. Further retrospective studies may help to determine the usefulness of NP-C SI models in clinical practice and refine their use.

## Conclusions

This study demonstrated the applicability of use of various SI models in clinical practice for screening and identification of patients with NP-C compared with clinical diagnosis. The study also demonstrated that the use of SI models can help physicians to identify suspected cases of NP-C for further clinical investigation. Of the available SI models, the Refined SI outperformed other models in identifying NP-C in patients prior to clinical diagnosis. The study confirms that SI models are useful screening tools that might facilitate the identification of patients with NP-C earlier in their disease course.

## Additional files

Additional file 1: **Figure S1.** The Original SI**.** Symptoms are scored according to their relative association with positive NP-C diagnosis. The combination of symptoms and the patient’s family history together provide the prediction score. NP-C, Niemann-Pick disease Type C; SI, Suspicion Index. (PDF 619 kb)

Additional file 2: **Figure S2.** The Early-Onset SI. Symptoms were scored based on the strength of association with a diagnosis of NP-C in the patient cohort. The CNS signs ataxia and mental regression contribute additional risk prediction score points only in the presence of other CNS signs (gelastic cataplexy, VSGP) or splenomegaly. CNS, central nervous system; NP-C, Niemann-Pick disease Type C; SI, Suspicion Index; VSGP, vertical supranuclear gaze palsy. (PDF 584 kb)

Additional file 3: **Figure S3.** Symptoms in patients diagnosed at clinic earlier than or the same time as the SIs**.** NP-C, Niemann-Pick disease Type C; SI, Suspicion Index; VSGP, vertical supranuclear gaze palsy. (PDF 4168 kb)

Additional file 4: **Table S1.** Signs and symptoms evaluated in the 2/7 and 2/3 SI models and their associated scores. SI, Suspicion Index. (DOCX 16 kb)

## Data Availability

The datasets supporting the conclusions of this article are included within the article (and its additional files).

## Abbreviations

NP-C: Niemann-Pick disease Type C
SD: standard deviation
SI: Suspicion Index
VSGP: vertical supranuclear gaze palsy

## Footnotes

1 Miglustat is indicated for the treatment of progressive neurologic manifestations in adult and pediatric patients with NP-C. Miglustat is approved for NP-C under the brand name Zavesca® in European Union/European Economic Area, Canada, Switzerland, Brazil, Australia, Iceland, Iran, Liechtenstein, Norway, Turkey, South Korea, New Zealand, Russia, Colombia, Mexico, Argentina, Chile, Ecuador, Israel, Palestine, Panama, Peru, Venezuela and Thailand. In Japan and China, miglustat is approved for NP-C under the brand name Brazaves®. Miglustat is not approved for the treatment of NP-C in the US and Taiwan.

## References

[1] Geberhiwot T, Moro A, Dardis A, Ramaswami U, Sirrs S, Marfa MP (2018). Consensus clinical management guidelines for Niemann-pick disease type C. Orphanet Journal of Rare Diseases.

[2] Mengel E, Pineda M, Hendriksz CJ, Walterfang M, Torres JV, Kolb SA (2017). Differences in Niemann-pick disease type C symptomatology observed in patients of different ages. Mol Genet Metab.

[3] Pineda M, Mengel E, Jahnová H, Héron B, Imrie J, Lourenço CM (2016). A suspicion index to aid screening of early-onset Niemann-pick disease type C (NP-C). BMC Pediatr.

[4] Wraith JE, Sedel F, Pineda M, Wijburg FA, Hendriksz CJ, Fahey M (2014). Niemann-pick type C suspicion index tool: analyses by age and association of manifestations. J Inherit Metab Dis.

[5] Patterson MC, Mengel E, Wijburg FA, Muller A, Schwierin B, Drevon H (2013). Disease and patient characteristics in NP-C patients: findings from an international disease registry. Orphanet J Rare Dis..

[6] Patterson MC, Clayton P, Gissen P, Anheim M, Bauer P, Bonnot O, et al. Recommendations for the detection and diagnosis of Niemann-pick disease type C: an update. Neurol Clin Pract 2017;7:499–511. 10.1212/CPJ.0000000000000399 PMC5800709 29431164

[7] Karimzadeh P, Tonekaboni SH, Ashrafi MR, Shafeghati Y, Rezayi A, Salehpour S (2013). Effects of miglustat on stabilization of neurological disorder in niemann-pick disease type C: Iranian pediatric case series. J Child Neurol.

[8] Pineda M, Wraith JE, Mengel E, Sedel F, Hwu WL, Rohrbach M (2009). Miglustat in patients with Niemann-pick disease type C (NP-C): a multicenter observational retrospective cohort study. Mol Genet Metab.

[9] Abe K, Sakai N. Patient with Niemann-pick disease type C: over 20 years' follow-up. BMJ Case Rep. 2017;2017. 10.1136/bcr-2017-220134. 10.1136/bcr-2017-220134 PMC5613829 28830896

[10] Di Rocco M, Dardis A, Madeo A, Barone R, Fiumara A (2012). Early miglustat therapy in infantile Niemann-pick disease type C. Pediatr Neurol.

[11] Fecarotta S, Romano A, Della Casa R, Del Giudice E, Bruschini D, Mansi G (2015). Long term follow-up to evaluate the efficacy of miglustat treatment in Italian patients with Niemann-pick disease type C. Orphanet J Rare Dis..

[12] Wijburg FA, Sedel F, Pineda M, Hendriksz CJ, Fahey M, Walterfang M (2012). Development of a suspicion index to aid diagnosis of Niemann-pick disease type C. Neurology..

[13] Hendriksz CJ, Pineda M, Fahey M, Walterfang M, Stampfer M, Runz H, Patterson MC, Torres JV, Kolb SA. The Niemann-pick disease type C suspicion index: development of a new tool to aid diagnosis. J Rare Dis Diagn Ther. 2015;1. 10.21767/2380-7245.100011.

[14] Synofzik M, Fleszar Z, Schöls L, Just J, Bauer P, Torres Martin JV (2016). Identifying Niemann-pick type C in early-onset ataxia: two quick clinical screening tools. J Neurol.

[15] Pineda M, Jurickova K, Karimzadeh P, Kolnikova M, Malinova V, Insua JL (2019). Disease characteristics, prognosis and miglustat treatment effects on disease progression in patients with Niemann-pick disease type C: an international, multicenter, retrospective chart review. Orphanet J Rare Dis.

[16] Pineda M, Perez-Poyato MS, O'Callaghan M, Vilaseca MA, Pocovi M, Domingo R (2010). Clinical experience with miglustat therapy in pediatric patients with Niemann-pick disease type C: a case series. Mol Genet Metab.

[17] Iturriaga C, Pineda M, Fernandez-Valero EM, Vanier MT, Coll MJ (2006). Niemann-pick C disease in Spain: clinical spectrum and development of a disability scale. J Neurol Sci.

[18] Strupp M, Kremmyda O, Adamczyk C, Bottcher N, Muth C, Yip CW (2014). Central ocular motor disorders, including gaze palsy and nystagmus. J Neurol.

[19] Wassif CA, Cross JL, Iben J, Sanchez-Pulido L, Cougnoux A, Platt FM (2016). High incidence of unrecognized visceral/neurological late-onset Niemann-pick disease, type C1, predicted by analysis of massively parallel sequencing data sets. Genet Med.

