# Supplementary material for: Evaluation of different suspicion indices in identifying patients with Niemann-Pick disease Type C in clinical practice: a post hoc analysis of a retrospective chart review
Source: Orphanet J Rare Dis. 2019 Jul 2;14:161. doi: 10.1186/s13023-019-1124-3 (PMC6604407; doi:10.1186/s13023-019-1124-3)
Supplement: Supplementary file 1 — Figure S1. The Original SI. Symptoms are scored according to their relative association with positive NP-C diagnosis. The combination of symptoms and the patient’s family history together provide the prediction score. NP-C, Niemann-Pick disease Type C; SI, Suspicion Index. (PDF 619 kb) [file 13023_2019_1124_MOESM1_ESM.pdf]

Signs and symptoms

Indicators	Visceral	Score	Neurological	Score	Psychiatric	Score
**Very strong**<br>40 points per item			• Vertical supranuclear gaze palsy<br>• Gelastic cataplexy	☐<br>☐		
**Strong**<br>20 points per item	• Prolonged unexplained neonatal jaundice or cholestasis<br>• Isolated unexplained splenomegaly (historical and/or current) with/without hepatomegaly	☐<br>☐			• Pre-senile cognitive decline and/or dementia	☐
**Moderate**<br>10 points per item			• Ataxia, clumsiness or frequent falls<br>• Dysarthria and/or dysphagia<br>• Dystonia	☐<br>☐<br>☐	• Psychotic symptoms (hallucinations, delusions and/or thought disorder	☐
**Weak**<br>5 points per item			• Acquired and progressive spasticity	☐	• Treatment-resistant psychiatric symptoms<br>• Other psychiatric disorders	☐<br>☐
**Ancillary**<br>1 point per item	• Hydrops fetalis<br>• Siblings with fatal ascites	☐<br>☐	• Hypotonia<br>• Delayed developmental milestones<br>• Seizure (partial or generalised)<br>• Myoclonus	☐<br>☐<br>☐<br>☐	• Disruptive or aggressive behaviour in adolescence and childhood	☐

Category scores

☐ + ☐ + ☐

Category Combination
40 points: Visceral & psychiatric
40 points: Visceral & neurological
20 points: Neurological & psychiatrics

Visceral & psychiatric ☐ + Visceral & neurological ☐ + Neurological & psychiatric ☐

NP-C family relationship
40 points: Parent/sibling
10 points: Cousin

Parent or sibling with NP-C ☐ + Cousin with NP-C ☐

Risk Prediction Score

☐ = Sum of scores
