# Supplementary figures and images for: Evaluation of different suspicion indices in identifying patients with Niemann-Pick disease Type C in clinical practice: a post hoc analysis of a retrospective chart review
Source: Orphanet J Rare Dis. 2019 Jul 2;14:161. doi: 10.1186/s13023-019-1124-3 (PMC6604407; doi:10.1186/s13023-019-1124-3)

**A) Original SI**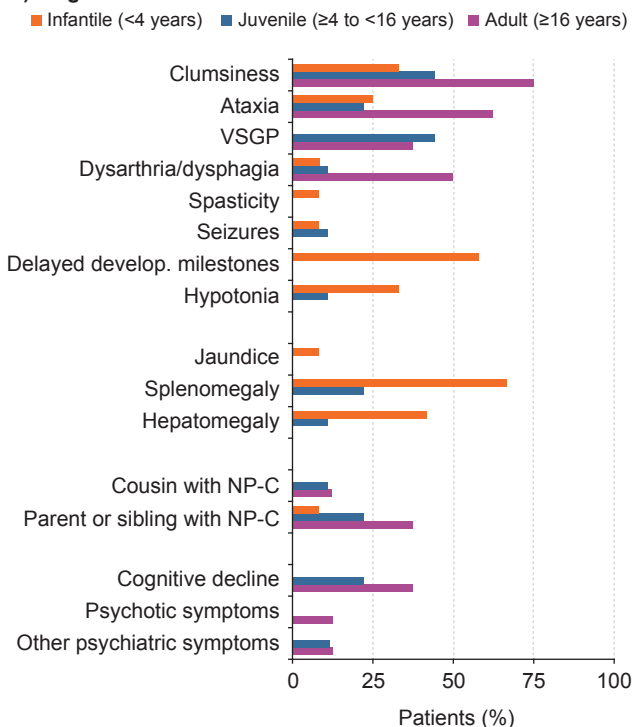**B) 2/7 SI**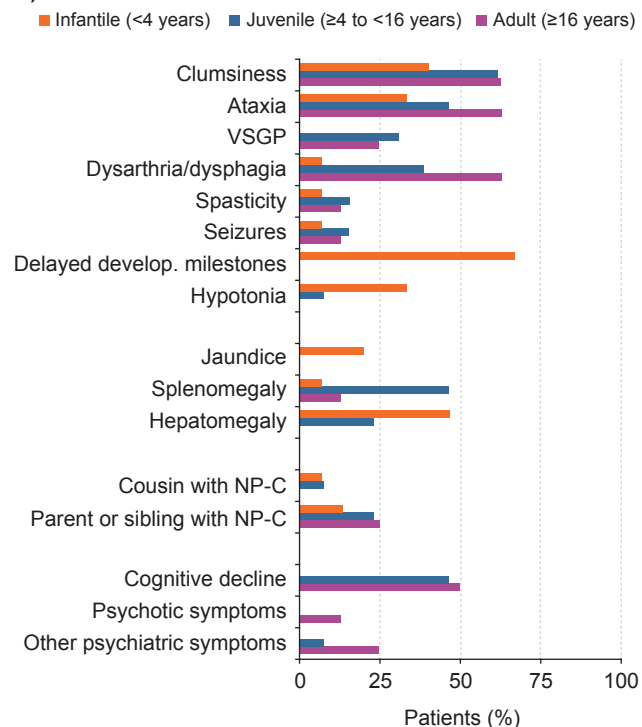**C) 2/3 SI**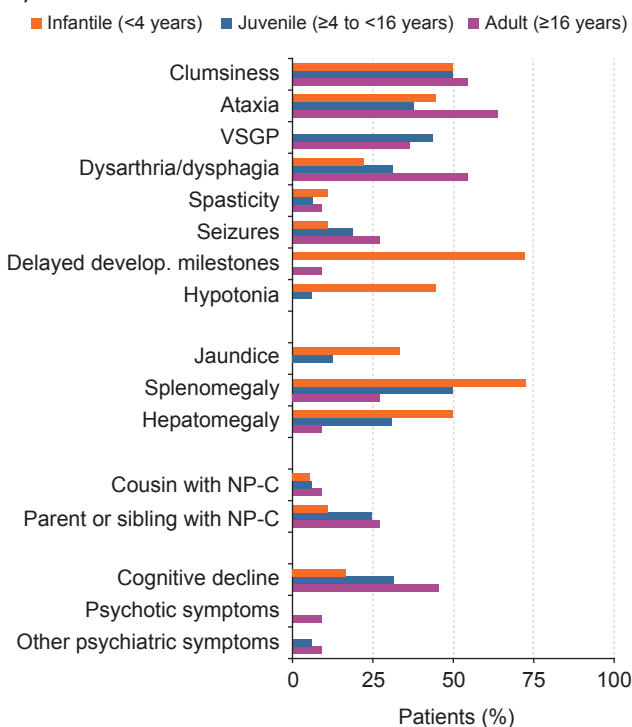**D) Early-Onset SI**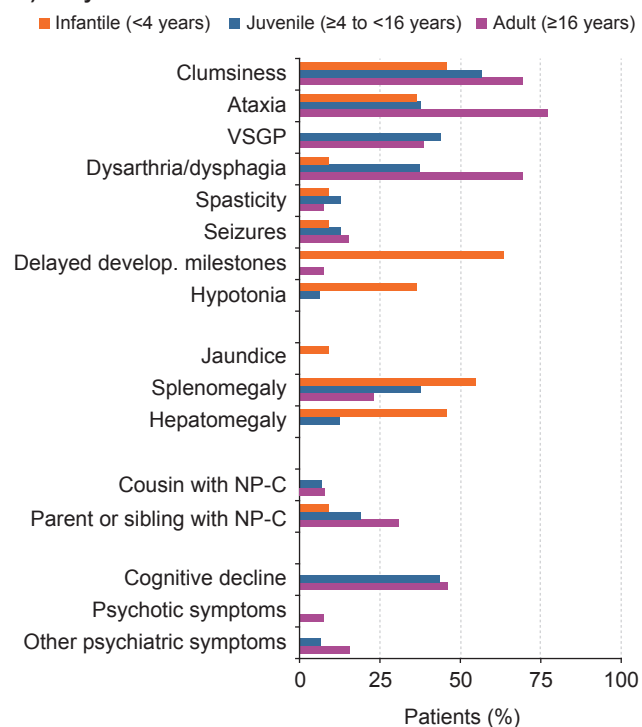

Supplement: Supplementary file 3 — Figure S3. Symptoms in patients diagnosed at clinic earlier than or the same time as the SIs. NP-C, Niemann-Pick disease Type C; SI, Suspicion Index; VSGP, vertical supranuclear gaze palsy. (PDF 4168 kb) [file 13023_2019_1124_MOESM3_ESM.pdf]
