# Supplementary material for: Evaluation of different suspicion indices in identifying patients with Niemann-Pick disease Type C in clinical practice: a post hoc analysis of a retrospective chart review
Source: Orphanet J Rare Dis. 2019 Jul 2;14:161. doi: 10.1186/s13023-019-1124-3 (PMC6604407; doi:10.1186/s13023-019-1124-3)
Supplement: Supplementary file 4 — Table S1. Signs and symptoms evaluated in the 2/7 and 2/3 SI models and their associated scores. SI, Suspicion Index. (DOCX 16 kb) [file 13023_2019_1124_MOESM4_ESM.docx]

**Supplementary Table 1** Signs and symptoms evaluated in the 2/7 and 2/3 SI models and their associated scores.

| **Signs and symptoms** | **Points** |
| --- | --- |
| **2/7 SI** | |
| Prolonged, unexplained neonatal jaundice or cholestasis | 1 |
| Isolated unexplained splenomegaly (historical and/or current) with or without hepatomegaly | 1 |
| Vertical supranuclear gaze palsy | 2 |
| Gelastic cataplexy | 1 |
| Pre-senile cognitive decline and/or dementia | 1 |
| Psychotic symptoms (hallucinations, paranoid delusions and/or thought disorder) | 1 |
| Parent, sibling or cousin with NP-C (two items combined) | 1 |
| **2/3 SI** | |
| Vertical supranuclear gaze palsy | 1 |
| Pre-senile cognitive decline and/or dementia | 1 |
| Dystonia | 1 |

SI, Suspicion Index
