# Supplementary material for: Evaluation of different suspicion indices in identifying patients with Niemann-Pick disease Type C in clinical practice: a post hoc analysis of a retrospective chart review
Source: Orphanet J Rare Dis. 2019 Jul 2;14:161. doi: 10.1186/s13023-019-1124-3 (PMC6604407; doi:10.1186/s13023-019-1124-3)
Supplement: Supplementary file 2 — Figure S2. The Early-Onset SI. Symptoms were scored based on the strength of association with a diagnosis of NP-C in the patient cohort. The CNS signs ataxia and mental regression contribute additional risk prediction score points only in the presence of other CNS signs (gelastic cataplexy, VSGP) or splenomegaly. CNS, central nervous system; NP-C, Niemann-Pick disease Type C; SI, Suspicion Index; VSGP, vertical supranuclear gaze palsy. (PDF 584 kb) [file 13023_2019_1124_MOESM2_ESM.pdf]

Points	CNS	Spleen	Liver	Lung	Natal	Family history
+4		Splenomegaly		Pulmonary infiltrates		
+3	Gelastic cataplexy		Prolonged jaundice			Parents or siblings with NP-C
+2	VSGP		Direct bilirubinaemia		Foetal oedema or ascites	
+1			Hepatomegaly			

**Modifiers** Add if presence of	CNS signs	Splenomegaly	Family history
+2	Atexia		
+1	Mental regression	Ataxia<br>Mental regression	
			+6

TOTAL
